# A novel pore-region mutation, c.887G > A (p.G296D) in *KCNQ4*, causing hearing loss in a Chinese family with autosomal dominant non-syndromic deafness 2

**DOI:** 10.1186/s12881-017-0396-5

**Published:** 2017-03-24

**Authors:** Bangqing Huang, Yanping Liu, Xue Gao, Jincao Xu, Pu Dai, Qingwen Zhu, Yongyi Yuan

**Affiliations:** 1 Department of Otolaryngology, Chinese PLA General Hospital, Beijing, 100853 China; 2 Department of Otolaryngology, Hainan Branch of PLA General Hospital, Sanya, 572013 China; 3 Department of Otorhinolaryngology, General Hospital of the Rocket Force, Beijing, 100088 China; 4 Department of Otolaryngology, The Second Hospital Of Hebei Medical University, Shijiazhuang, 050018 China

**Keywords:** *KCNQ4*, Autosomal dominant non-syndromic deafness 2, Novel mutation, Next-generation sequencing

## Abstract

**Background:**

Hereditary non-syndromic hearing loss is the most common inherited sensory defect in humans. The KCNQ4 channel belongs to a family of potassium ion channels that play crucial roles in physiology and disease. Mutations in *KCNQ4* underlie deafness non-syndromic autosomal dominant 2, a subtype of autosomal dominant, progressive, high-frequency hearing loss.

**Methods:**

A six-generation Chinese family from Hebei Province with autosomal dominantly inherited, sensorineural, postlingual, progressive hearing loss was enrolled in this study. Mutation screening of 129 genes associated with hearing loss was performed in five family members by next-generation sequencing (NGS). We also carried out variant analysis on DNA from 531 Chinese individuals with normal hearing as controls.

**Results:**

This family exhibits postlingual, progressive, symmetrical, bilateral, non-syndromic sensorineural hearing loss. NGS, bioinformatic analysis, and Sanger sequencing confirmed the co-segregation of a novel mutation [c.887G > A (p.G296D)] in *KCNQ4* with the disease phenotype in this family. This mutation leads to a glycine-to-aspartic acid substitution at position 296 in the pore region of the KCNQ4 channel. This mutation affects a highly conserved glutamic acid. NGS is a highly efficient tool for identifying gene mutations causing heritable disease.

**Conclusions:**

Progressive hearing loss is common in individuals with *KCNQ4* mutations. NGS together with Sanger sequencing confirmed that the five affected members of this Chinese family inherited a missense mutation, c.887G > A (p.G296D), in exon 6 of *KCNQ4*. Our results increase the number of identified *KCNQ4* mutations.

**Electronic supplementary material:**

The online version of this article (doi:10.1186/s12881-017-0396-5) contains supplementary material, which is available to authorized users.

## Background

Hearing loss is a very common sensory defect and a highly heterogeneous sensory disorder in humans. The majority of congenital cases of hearing loss are attributable to genetic factors. Hereditary hearing loss can be classified according to the pattern of inheritance, and the presence (syndromic) or absence (non-syndromic) of distinctive clinical features. The inheritance patterns of non-syndromic hearing loss (NSHL) include autosomal dominant, autosomal recessive, X-linked, and mitochondrial inheritance. Autosomal dominant deafness accounts for approximately 20% of cases of hereditary hearing loss [1]. Autosomal dominant non-syndromic hearing loss (ADNSHL) has extremely heterogeneous genetic and clinical features. To date, more than 60 loci for ADNSHL have been mapped to chromosomal regions and 27 genes for non-syndromic deafness, autosomal dominant (DFNA) have been identified (http://hereditaryhearingloss.org). Recently, high-throughput sequencing, involving targeted sequencing of the protein-coding subset of the human genome, has become a highly efficient tool due to its ability to perform parallel sequencing of millions of nucleotides at relatively low cost and high speed. This should greatly improve the screening of thousands of target genes, making this an ideal technique for identifying causative genes and mutations involved in heritable hearing disease [2].

Based on the number of reported mutations, *KCNQ4* (the gene responsible for DFNA2) is one of the genes most commonly responsible for ADNSHL [3]. KCNQ4 (voltage-gated potassium channel, KQT-like subfamily Q, member 4), the first identified causal gene of ADNSHL at the DFNA2 locus, was discovered and cloned by Kubisch in 1999 [4]. *KCNQ4* was mapped to 1p34, within the DFNA2 locus; *KCNQ4* is a member of the voltage-gated potassium channel family and plays a pivotal role in potassium recycling in the inner ear. Its cDNA encodes a polypeptide of 695 amino acids that forms a voltage-gated potassium Kv7.4 channel protein. *KCNQ4*, together with *KCNQ1*, *KCNQ2*, and *KCNQ3*, constitutes a distinct branch of the superfamily of voltage-gated channels [4].

These voltage-gated channels typically contain four subunits that encircle a central pore, which enables the selective passage of potassium ions across the cell membrane. Each subunit consists of six transmembrane segments (S1–S6) encoded by six exons (exons 2 to 7), with both N- and C-termini being located on the intracellular side of the membrane. The S4 segment comprises the voltage sensor of the channel; and the S5 and S6 portions, connected with an intervening re-entrant loop (P-loop domain), form the pore region. Four P-loop domains combine to form the selectivity filter of the channel [5]. KCNQ4 is expressed in sensory hair cells of the inner ear and in the central auditory pathway, defects in *KCNQ4* caused hearing loss by a slow degeneration of outer hair cells resulting from chronic depolarization [6]. Autosomal dominant non-syndromic hearing loss causing by *KCNQ4* mutations usually starts from high-frequency. Most of the missense mutations identified to date are located in the pore region of the KCNQ4 channel, namely, the P-loop domain [4]. Missense mutant in pore region, e.g. p.G285S and p.G296S, exerts a strong dominant-negative effect on potassium currents by reducing the wild type KCNQ4 channel expression at the cell surface, causing a greater reduction of KCNQ4 current to the cell membrane [4, 7]. In this study, we report the genetic basis of ADSHNL in a Chinese family, as determined by NGS together with Sanger sequencing, and identify a novel missense mutation, c.887G > A (p.G296D), in the pore region of the KCNQ4 channel.

## Methods

### Family members and clinical evaluations

**Fig. 1:**
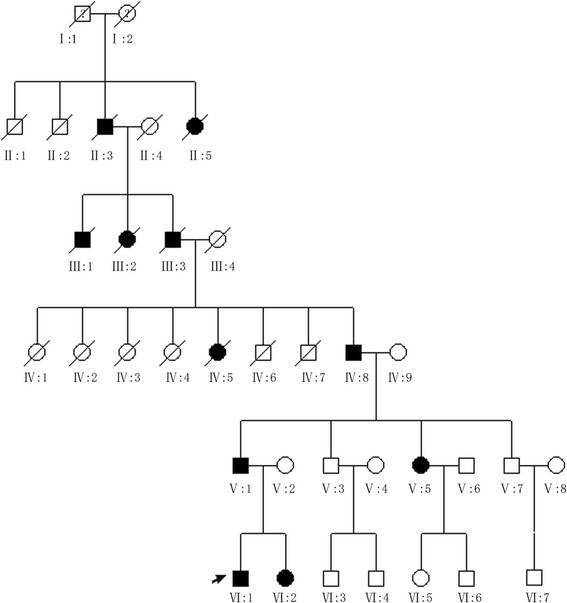
Pedigree of the Chinese DFNA family HBJ. Affected family members are denoted in black. The arrow indicates the proband

The family, referred to here as HBJ, is a six-generation Chinese family with 35 members of Han origin from Hebei Province with autosomal dominant, postlingual, progressive, non-syndromic sensorineural hearing loss (Fig. 1). Eight members of this family participated in our study, including five affected, and three unaffected. Medical histories of the members of the family were obtained via a questionnaire on the following aspects of this condition: subjective degree of hearing loss (the clinical history ruled out environmental factors as the cause of hearing loss), age at onset, progression, symmetry of the hearing impairment, use of aminoglycosides, presence of tinnitus, use of hearing aids, noise exposure, medication, pathological changes in the ear, and other relevant clinical manifestations. Physical examinations ruled out the possibility of syndromic hearing loss. Audiometric evaluations and otological examinations included otoscopy, pure tone audiometry (PTA), acoustic immittance measurement, auditory brainstem responses, and distortion product otoacoustic emissions (DPOAE). PTA was calculated as the average of the thresholds measured at 0.5, 1.0, 2.0, and 4.0 kHz, and performed to test for air conduction (125–8000 Hz) and bone conduction (250–4000 Hz). The severity of hearing impairment was defined as mild (26–40 dB), moderate (41–55 dB), moderately severe (56–70 dB), severe (71–90 dB), or profound (>90 dB). Tympanometry indicated proper functioning of the middle ear. A high-resolution computed tomography (HRCT) scan of the temporal bone was performed on some of the affected individuals. The diagnosis of profound sensorineural hearing impairment was made in accordance with the ICD-10 (International Classification of Diseases 10th Revision) criteria based on audiometric examination.

### DNA extraction

Genomic DNA from eight subjects in the HBJ family and 531 Han Chinese with normal hearing was extracted from peripheral blood leukocytes using a blood DNA extraction kit (Qiagen, Hilden, Germany), in accordance with the manufacturer’s instructions. Ultraviolet spectrophotometry was used to measure the DNA concentration and purity.

### Screening for mutations in common deafness-related genes

Screening for mutations in common deafness-related genes was conducted using polymerase chain reaction (PCR) amplification and direct sequencing of exons. These included *GJB2*, *SLC26A4*, and mitochondrial *12S rRNA*; the primers and PCR conditions were as described in detail in our previous paper [8]. The results of screening of these common deafness-related genes were all negative.

### Deafness gene capture and Illumina library preparation

Mutation screening of 129 genes associated with hearing loss was performed in five family members by NGS. Deafness genes capture and Illumina library preparation were performed as the description in our previous paper [9, 10].

### Sanger sequencing

After filtering against multiple databases, Sanger sequencing was used to determine whether any of the potential mutations in known genes causing ADNSHI cosegregated with the phenotype in this family. Direct PCR products were sequenced using Bigdye terminator v3.1 cycle sequencing kits (Applied Biosystems, Foster City, CA, USA) and analysed using an ABI 3700XL Genetic Analyzer.

### Mutational analysis

Segregation of the mutations was evaluated in the family. Genotyping for c.887G > A was performed by PCR and detected by bidirectional sequencing of the amplified fragments using an automated DNA sequencer (ABI3100); the primers were 5′-GAATCCATCTATGACCCTAACCA-3′ and 5′- GCTTCTCGAAGTGCTTCTGC-3′. Nucleotide alterations were identified by sequence alignment with the *KCNQ4* GenBank sequence (NM_014208) using Genetool software.

### Multiple sequence alignment

Multiple sequence alignment was performed across 15 species using Clustal Omega (http://www.ebi.ac.uk/Tools/msa/clustalo/).

### Model building and structure-based analysis

Three-dimensional modeling of the human wild-type and p.G296D mutant of KCNQ4 was performed using SWISSMODEL. In this study, the automatic modeling approach was applied to the complete protein sequence of human KCNQ4, including its 695 amino acids, and its mutant, which are available in NCBI GenBank (NP_004691.2) in FASTA format. Data obtained by the homology models were visualized using Swiss-PdbViewer 4.1.

## Results

### Clinical evaluations

**Fig. 2:**
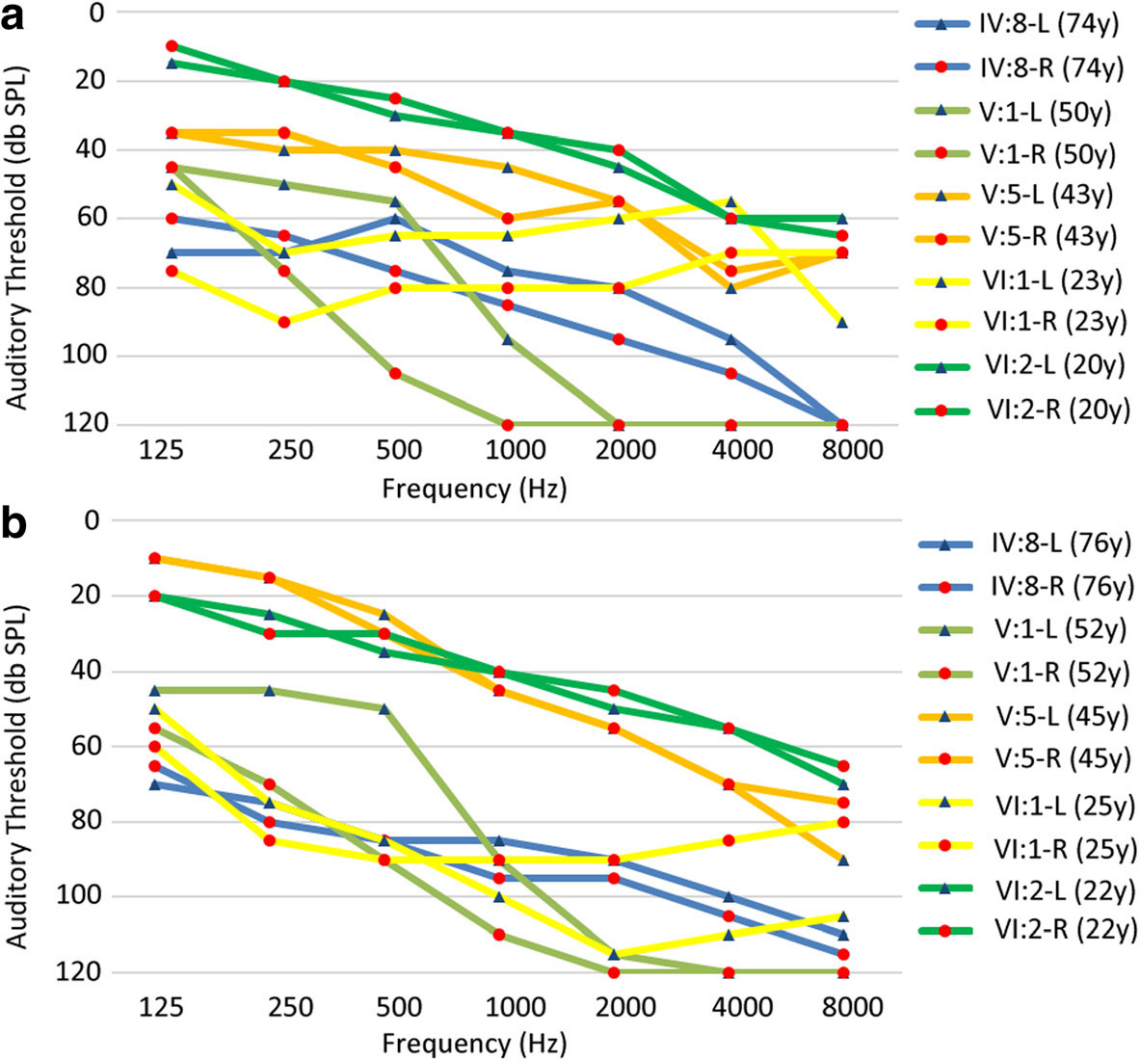
The audiometric curves of the affected members in HBJ. **a** At the beginning of the study in 2014; **b** Follow up in 2016

In the HBJ family, 5 clinically affected and 12 unaffected individuals were identified. The age at onset of hearing impairment ranged from 15 to 30 years. The audiological assessments and clinical history of the affected members in this family showed postlingual, progressive, symmetrical, bilateral, non-syndromic, sensorineural hearing loss. The audiogram patterns of the patients were distinct; most of them initially showed high-frequency hearing loss, but in the proband (VI:1), hearing loss at all frequencies was seen from the outset. The hearing loss initially involved high frequencies, with subsequent gradual progression to a severe level involving all frequencies. The subjects with hearing loss also reported tinnitus, but there were no vestibular signs or symptoms (Table 1, Fig. 2).

**Table 1:** Phenotypes and genotypes of the family members in this study

Family members	Age of onset (years)	Nucleotide change	At the beginning of this study (2014)				Follow-up (2016)				Tinnitus (No or Yes)	Exposure to noise (No or Yes)	Ototoxic drugs expose (No or Yes)	Vertigo (No or Yes)
			PTA		DPOAE		PTA		DPOAE					
			Right	Left	Right	Left	Right	Left	Right	Left				
IV:8	25	c.887G > A	90	77.5	Absent at all frequencies	Absent at all frequencies	95	90	Absent at all frequencies	Absent at all frequencies	Yes	No	No	No
V:1	15	c.887G > A	116.25	97.5	Absent at all frequencies	Absent at all frequencies	110	93.75	Absent at all frequencies	Absent at all frequencies	Yes	No	No	No
V:2	-	wildtype	15	12.5	Not examined	Not examined	20	15	Not examined	Not examined	No	No	No	No
V:3	-	wildtype	13.75	28.75	Not examined	Not examined	15	33.75	Normal	Normal at 1 and 2 KHz and absent at other frequencies	No	Yes	No	No
V:5	30	c.887G > A	58.75	55	Absent at all frequencies	Absent at all frequencies	50	48.75	Absent at all frequencies	Absent at all frequencies	Yes	No	No	No
V:7	-	wildtype	20	17.5	Not examined	Not examined	15	18.75	Normal	Normal	No	No	No	No
VI:1	18	c.887G > A	77.5	61.25	Absent at all frequencies	Absent at all frequencies	88.75	103.8	Absent at all frequencies	Absent at all frequencies	Yes	No	No	No
VI:2	21	c.887G > A	40	42.5	Not examined	Not examined	42.5	45	Not examined	Not examined	Yes	No	No	No

An analysis of the temporal bone scans of the affected members by HRCT showed a normal middle-ear structure, including normal internal auditory canal and vestibular aqueduct. DPOAE testing showed cochlear dysfunction in the patients.

### Candidate gene analyses

**Fig. 3:**
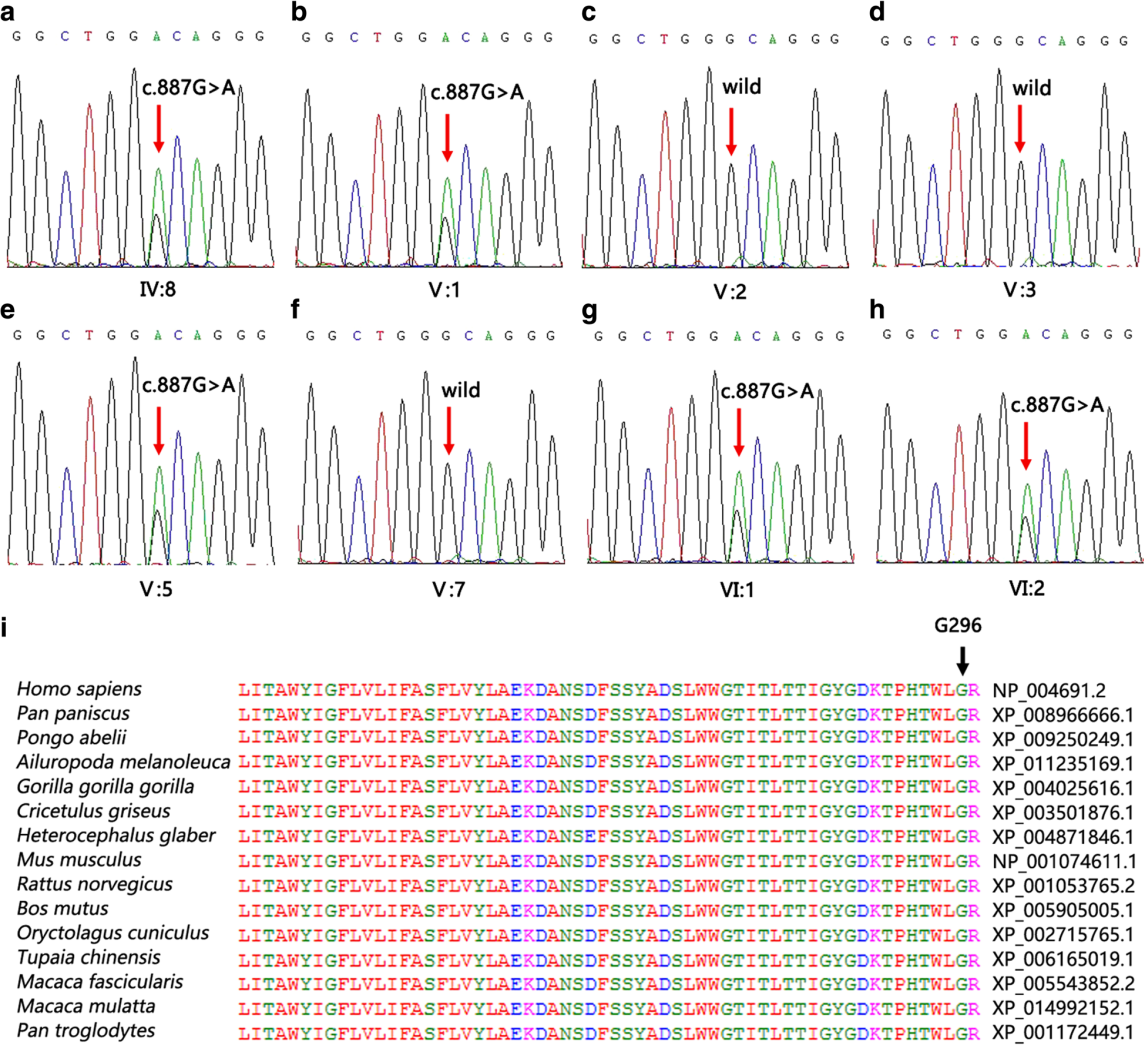
Mutation detection and conservation analysis. **a** ~ **h** The sanger sequence results of the family members; **i** Protein alignment shows conservation of the G296 residue of KNCQ4 across 15 species

After the filtering process, we found that variants in *MYO7A, GJB2, MITF, DIAPH1, PDZD7, WFS1, TRIOBP, GPR98, MYO1A, MYO15A* and *KCNQ4* might be the potential mutations. For details please see Additional file 1: Table S1. However, only the variant in *KCNQ4* co-segregated within the family. We identified a novel mutation [c.887G > A (p.G296D)] in exon 6 of *KCNQ4* in the five affected family members. This mutation results in a glycine to aspartic acid substitution at position 296 in KCNQ4. Sanger sequencing revealed that all of the affected family members were heterozygous for this mutation, which was not present in the unaffected family members (Fig. 3a ~ h). The *KCNQ4* c.887G > A mutation was also not detected in the normal hearing controls. The depth and coverage information for the DFNA2 locus was provided in Additional file 2: Table S2.

The glycine at position 296 is conserved across 15 species, as depicted in Fig. 3i. PROVAN, SIFT, PolyPhen, Mutation Taster and MutationAssessor predicted that *KCNQ4* c.887G > A (p.G296D) would be a detrimental mutation (Table 2). In terms of protein structure, glycine is highly conserved in the K^+^ channels of 15 different species, and is completely conserved among members of the KCNQ family, as well as of the super-family of voltage-gating K^+^ channels.

**Table 2:** Pathogenicity Assessment in Silico of *KCNQ4* c.887G > A (p.G296D)

Tools	Pathogenicity	Functional Prediction Scores/Conservation scores
PROVEAN	Deleterious	−6.558
SIFT	Damaging	0
PolyPhen	Probably damaging	1
Mutation Taster	Disease causing	1
MutationAssessor		4.39

### Structural modeling of p.G296D

**Fig. 4:**
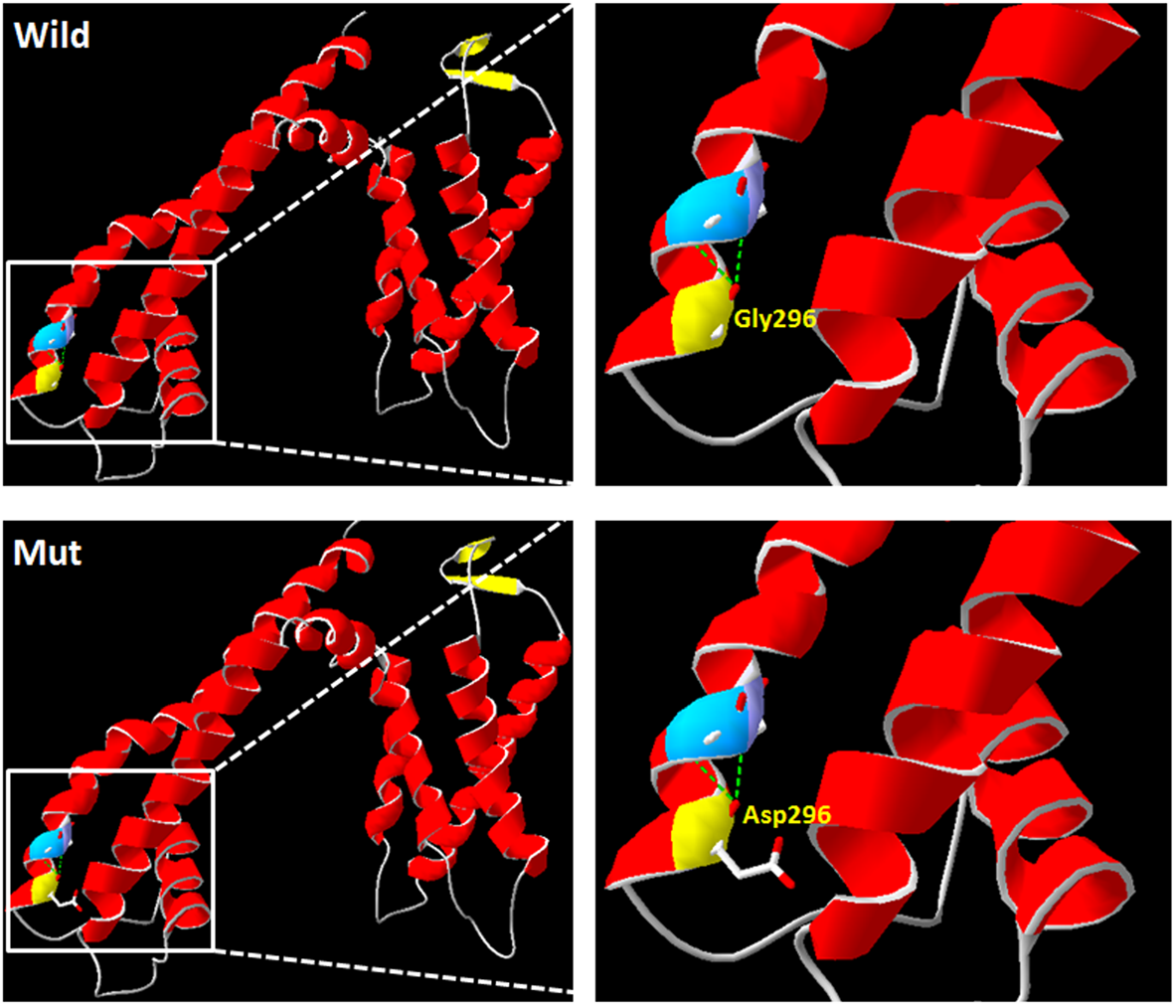
Structure of wild-type and mutant 296 of KCNQ4. Upper panel: wild-type G296; lower panel: mutant D296

A structural model of the p.G296D variant of *KCNQ4* was constructed based on the crystal structure of 4chvA. The model covered the target sequence of KCNQ4 (residues 101–335). Swiss-PdbViewer 4.1 predicted that this mutation perturbs the amino acid side chain because of the replacement of glycine by aspartic acid. Both wild-type Glycine and mutant-type Aspartic forms two salt bridges with Leucine at position 299 and Alanine at position 300 (Fig. 4). Since the sequence identity between the target and the template was only 22.46%, there might be other protein structure change that were absent in this model.

## Discussion

The *KCNQ4* gene encodes potassium voltage-gated channel subfamily KQT member 4, which is expressed in sensory outer hair cells (OHCs) [4]. *KCNQ4* is linked to the DFNA2 locus on chromosome 1p34, which is thought to play a critical role in the regulation of neuronal excitability, particularly in sensory cells of the cochlea. Mutations in *KCNQ4* have been shown to be associated with ADNSHL, mainly by impairing the function of OHCs [4]. To date, 20 mutations in *KCNQ4* have been reported to cause hearing loss. All are located in exons 1, 4–8 of *KCNQ4* (Table 3) [4, 7, 11–25].

**Table 3:** Overview of *KCNQ4* mutations described in DFNA2

Mutation	Protein change	Exon	Origin	Age of onset	Sub-domain	Reference
c.211delC	p.Q71fs	1	Japan	8–50	N-term cyto	[17, 18]
c.211del13	p.Q71fs	1	Belgian	<10	N-term cyto	[15]
c.229_230insGC	p.H77fs	1	Japan	27	N-term cyto	[18]
c.546C > G	p.F182L	4	Japan	--	S3 trans	[18, 19]
c.664_681del18	p.G215_220del6	4	Korea	earliest 4	S4–S5 linker	[14]
c.689 T > A	p.V230E	4	Japan	3–40	S4–S5 linker	[18]
c.725G > A	p.W241X	5	America	--	S5 trans	[16]
c.778G > A	p.E260K	5	America	--	S5 trans	[16]
c.785A > T	p.D262V	5	America	--	S5 trans	[16]
c.806_808delCCT	p.S269del	5	Canada	<10	S5–S6 linker	[11]
c.821 T > A	p.L274H	5	Neth	--	PR(P)	[23]
c.823 T > C	p.W275R	5	China	2–30	PR(P)	[24]
c.827G > C	p.W276S	5	Japan,Dutch	<10	PR(P)	[12, 15, 21, 22]
c.842 T > C	p.L281S	6	America	--	PR(P)	[20]
c.853G > T	p.G285C	6	America	<10	PR(P)	[15]
c.853G > A	p.G285S	6	French,China	6–30	PR(P)	[4, 24]
c.859G > C	p.G287R	6	America	1–21	PR(P)	[13]
c.871C > T	p.P291S	6	Japan	20	PR(P)	[18]
c.872C > T	p.P291L	6	Japan	17	PR(P)	[18]
c.886G > A	p.296S	6	Spanish	9–50	PR	[7]
c.887G > A	p.G296D	6	China	15–25	PR	This study
c.891G > T	p.R297S	6	Japan	5,39	S6 trans	[18]
c.961G > A	p.G321S	7	Dutch	<10	S6 trans	[15]
c.1044_1051del8	p.A349Pfs	8	Japan	<10	S6-B segment linker	[25]

Abbreviations: *cyto* cytoplasmic, *trans* transmembrane, *PR* Pore region, *(P)* P-loop

According to previous reports, mutations in *KCNQ4* exhibit autosomal dominant inheritance. However, Wasano et al. recently identified another novel *KCNQ4* mutation, c.1044_1051del8 (p.A349Pfs), in a family with autosomal recessive NSHL [25]. This suggests that mutation in *KCNQ4* can cause autosomal recessive hearing loss with a more severe phenotype than autosomal dominant hearing loss.

Using NGS as well as Sanger sequencing, we identified a novel mutation, c.887G > A (p.G296D), which results in a glycine-to-aspartic acid substitution at position 296 in the pore region of KCNQ4. This mutation co-segregated with the phenotype in the family and was not detected in normal hearing controls.

The novel missense mutation p.G296D is in the pore region of KCNQ4, where also resides the mutations p.L274H [23], p.W275R [24], p.W276S [12, 15, 21, 22], p.L281S [20], p.G285S [4, 24], p.G285C [4], p.G287R [13], p.P291S [18], p.P291L [18] and p.G296S [7]. In a previous study, Mencia et al. reported that the mutation c.886G > A (p.G296S) in *KCNQ4* is pathogenic [7]. Mencia et al. demonstrated both effects of the mutation: reduced surface expression and abolished channel function. We speculate that the p.G296D mutation may affect the function of KCNQ4 channel through the mechanism that the mutation of amino acid residues in a single subunit of a tetrameric channel can block the permeation pathway and inhibit the current, leading to depolarization and death of outer hair cells [4, 6, 26].

The age of onset of hearing loss, which ranges from the first to the fifth decade of life, differs among those with different mutations in *KCNQ4*. The age of onset of hearing loss caused by p.Q71fs in the N-terminal, p.G285C in the pore region, and p.A349Pfs in S6trans is in the first decade of life [15, 23, 25]. However, the average age of onset of hearing loss associated with the mutations p.W275R, p.G285S and p.G296S, located in the pore region, ranges from the first to the fourth decade [4, 7, 24]. In our study, the age of onset of hearing loss in the affected family members was in the second to third decades of life. In addition, the affected family members showed different hearing phenotypes. Most of the patients initially showed high-frequency hearing loss, while in the proband (VI-1), hearing of all frequencies was affected from the outset. This suggests that the same mutation in the same gene may have different effects among individuals in the same family. The present report, which is the first of p.G296D mutation in *KCNQ4*, adds to our understanding of *KCNQ4* mutation-induced hearing loss. This study also indicates that NGS is a valuable tool for the diagnosis of autosomal dominant deafness. We plan to follow the next generation of this family and hope to obtain further valuable information from them. In addition, we hope to provide them with more comprehensive genetic counseling, early diagnosis, and even treatment of hearing impairment.

## Conclusions

In this study, a novel *KCNQ4* mutation, c.887 G > A (p.G296D), was identified in all five affected members in a Chinese family with ADNSHL using NGS and Sanger sequencing. The hearing phenotype of the proband differed from that in previously reported pedigrees, and in other members of the same family with *KCNQ4* mutations. The same mutation in the same gene may thus have different effects among individuals in the same family. This may be related to the expressivity of the gene and the age of onset in the autosomal hereditary mode. The results of our study increase the content of databases of genes associated with genetic deafness.

## Additional files

Additional file 1: Table S1. Potential deafness causing variants found by NGS. (DOCX 14 kb)

Additional file 2: Table S2. Depth and coverage information of DFNA2 locus in NGS. (DOCX 14 kb)

## Abbreviations

ADNSHL: Autosomal dominant non-syndromic hearing loss
DFNA: Non-syndromic deafness autosomal dominant
DPOAE: Distortion product otoacoustic emissions
gDNA: Genomic DNA
HRCT: High-resolution computed tomography
NGS: Next-generation sequencing
NSHL: Non-syndromic hearing loss
PCR: Polymerase chain reaction
PTA: Pure tone audiometry
