# Supplementary material for: A novel pore-region mutation, c.887G > A (p.G296D) in KCNQ4, causing hearing loss in a Chinese family with autosomal dominant non-syndromic deafness 2
Source: BMC Med Genet. 2017 Mar 24;18:36. doi: 10.1186/s12881-017-0396-5 (PMC5366164; doi:10.1186/s12881-017-0396-5)
Supplement: Supplementary file 1 — Potential deafness causing variants found by NGS. (DOCX 14 kb) [file 12881_2017_396_MOESM1_ESM.docx]

**Supplemental Table 1. Potential deafness causing variants found by NGS**

|  | Gene | Variants | Family members carrying the variant | Cosegregation  Yes or No |
| --- | --- | --- | --- | --- |
| 1 | MYO7A | Het c.905G>A, p.R302H | Ⅳ8，Ⅴ7 | No |
| 2 | GJB2 | Het c.109G>A, p.V37I | Ⅳ8，Ⅴ2 | No |
| 3 | MITF | Hetc.160_161insA:p.R54fs Hetc.328_329insA:p.R110fs | Ⅳ8，Ⅴ2，Ⅴ5,Ⅴ7 | No |
| 4 | DIAPH1 | Het c.506G>T,p.S169I,  c. 533 G>T,p.S178I | Ⅳ8 | No |
| 5 | PDZD7 | Het c. 1267 G>A,p.A425T | Ⅴ1, Ⅴ2, Ⅴ5 | No |
| 6 | KCNQ4 | Het c.887G>A,p.G296D | Ⅳ8, Ⅴ1, Ⅴ5,  Ⅵ1 | Yes |
| 7 | TRIOBP | Het c. 2654 G>A,p.R885H | Ⅴ1 | No |
| 8 | GPR98, | Het c. 5620 A>G,p.S1874G | Ⅴ5，Ⅵ1 | No |
| 9 | MYO1A, | Het c. 2275 G>T,p.A759S | Ⅴ5 | No |
| 10 | MYO15A | Het c. 4741 A>T,p.T1581S | Ⅴ1, Ⅴ2，Ⅴ7 | No |
| 11 | WFS1 | Het c. 1153 G>A,p.E385K | Ⅴ1, Ⅴ7 | No |

Het heterozygous
