# Supplementary material for: A novel pore-region mutation, c.887G > A (p.G296D) in KCNQ4, causing hearing loss in a Chinese family with autosomal dominant non-syndromic deafness 2
Source: BMC Med Genet. 2017 Mar 24;18:36. doi: 10.1186/s12881-017-0396-5 (PMC5366164; doi:10.1186/s12881-017-0396-5)
Supplement: Supplementary file 2 — Depth and coverage information of DFNA2 locus in NGS. (DOCX 14 kb) [file 12881_2017_396_MOESM2_ESM.docx]

**Supplemental Table 2. Depth and coverage information of DFNA2 locus in NGS**

|  |  | **Ⅳ:8** | **Ⅴ:1** | **Ⅴ:2** | **Ⅴ:5** | **Ⅴ:7** | **Ⅵ:1** |
| --- | --- | --- | --- | --- | --- | --- | --- |
| **KCNQ4-14exons** | Exon_average_depth | 44.71 | 69.29 | 44.05 | 50.32 | 44.80 | 54.00 |
|  | Exon_coverage | 62.30% | 64.60% | 62.35% | 61.35% | 61.81% | 62.96% |
|  |  |  |  |  |  |  |  |
| **KCNQ4-exon6** | Exon_average_depth | 115.38 | 156.68 | 117.12 | 123.46 | 106.11 | 130.36 |
|  | Exon_coverage | 100.00% | 100.00% | 100.00% | 100.00% | 100.00% | 100.00% |
|  |  |  |  |  |  |  |  |
| **GJB3** | Exon_average_depth | 175.81 | 276.03 | 159.49 | 192.84 | 134.64 | 214.53 |
|  | Exon_coverage | 100.00% | 100.00% | 100.00% | 100.00% | 99.73% | 100.00% |
